# Necrotizing enterocolitis: Bench to bedside approaches and advancing our understanding of disease pathogenesis

**DOI:** 10.3389/fped.2022.1107404

**Published:** 2023-01-11

**Authors:** Dhirendra K. Singh, Claire M. Miller, Kelly A. Orgel, Mili Dave, Stephen Mackay, Misty Good

**Affiliations:** ^1^ Division of Neonatal-Perinatal Medicine, Department of Pediatrics, University of North Carolina at Chapel Hill, Chapel Hill, NC, United States; ^2^ University of North Carolina at Chapel Hill School of Medicine, Chapel Hill, NC, United States

**Keywords:** intestinal development, neonates, prematurity, necrotizing enterocolitis, intestinal epithelium

## Abstract

Necrotizing enterocolitis (NEC) is a devastating, multifactorial disease mainly affecting the intestine of premature infants. Recent discoveries have significantly enhanced our understanding of risk factors, as well as, cellular and genetic mechanisms of this complex disease. Despite these advancements, no essential, single risk factor, nor the mechanism by which each risk factor affects NEC has been elucidated. Nonetheless, recent research indicates that maternal factors, antibiotic exposure, feeding, hypoxia, and altered gut microbiota pose a threat to the underdeveloped immunity of preterm infants. Here we review predisposing factors, status of unwarranted immune responses, and microbial pathogenesis in NEC based on currently available scientific evidence. We additionally discuss novel techniques and models used to study NEC and how this research translates from the bench to the bedside into potential treatment strategies.

## Introduction

Necrotizing enterocolitis (NEC) is a gastrointestinal disease that commonly affects preterm infants and is a major cause of morbidity and mortality in neonatal intensive care units (NICUs). Despite the advancements made in providing neonatal intensive care in recent years, NEC remains a devastating disease in NICUs. Approximately 7%–8% of premature infants in the NICU are diagnosed with NEC, with mortality rates approaching 20%–30% (1, 2). Of those that survive, many suffer from detrimental long-term effects on the intestines, growth, and neurodevelopment (3, 4).

NEC is characterized by inflammation and necrosis in the intestines, and often presents with a distended abdomen and blood in the stool (5, 6). Currently, NEC is treated with either a medical or surgical approach. The medical approach for the milder stages of NEC, consists of cessation of feedings, stomach decompression, antibiotics, frequent monitoring, and supportive care. Surgery is required if the infant experiences gangrene or intestinal perforation, and this treatment approach carries a higher rate of mortality (7). These treatment approaches have not changed in several decades and novel approaches to prevent or treat NEC are desperately needed.

Research into identifying the etiology of NEC has revealed that the most prominent risk factor is infant prematurity (8, 9). Approximately 9 of 10 infants diagnosed with NEC are born premature (gestational ages 22–37 weeks), with the most severe cases typically manifesting in very low birth weight (VLBW) preterm infants with a birth weight of <1,500 grams. Although cases of NEC have been observed in full-term infants, VLBW infants maintain the highest chances of contracting and succumbing to NEC (10).

This increased occurrence and fatality in premature infants has been attributed in part to their underdeveloped innate and adaptive immune systems, as well as decreased diversity of the gut microbiome compared to those of full-term infants (11, 12). Research suggests that intestinal immaturity and undeveloped immunity of preterm infants allows pathogens to bypass the epithelial cell layer to induce inflammation (13). One of the ways to decrease NEC incidence is to provide maternal breast milk to infants. Human milk oligosaccharides (HMOs) and immunoglobins (Ig), such as immunoglobulin A (IgA), are present in breast milk and have been shown to protect against NEC (14, 15). The components in breast milk help prevent the onset of NEC and shift the infant's gut microbial composition, which in turn bolsters the immune response (16). While we have some idea of the factors that contribute to and the factors that protect against the disease, the specific mechanisms that lead to the pathogenesis of NEC are not fully understood.

In this review, we examine factors that may contribute to NEC and associated pathogenesis, including the role that the mucosal immune response and the microbiome play in disease. Furthermore, we outline the various *in vitro* and *in vivo* NEC models used to demonstrate these findings and explore how these conclusions can lead to the development of preventative measures and treatments designed for NEC.

## Factors that may contribute to NEC

Although the etiology of NEC has yet to be completely elucidated, there are a multitude of factors, before and after birth, that can predispose infants to NEC. Maternal health status can provide substantial insight into an infant's risk of contracting NEC. According to a review of NEC risk factors in infants, variables such as maternal age, intrapartum antibiotics, incomplete steroid exposure, and maternal high neutrophil to lymphocyte ratio (NLR) are significant prognostic factors (9). Several observational studies have examined these factors in detail. A retrospective case control study with 97 matched pairs of infants showed a significantly higher odds ratio for antenatal ampicillin exposure for infants who later developed NEC than control infants (17).

Considering antenatal steroid exposure, it has been established that this treatment reduces morbidities and improves overall neonatal survival. However, an incomplete course of antenatal steroids or no steroid exposure has been associated with higher rates of more severe NEC (18). In a separate retrospective cohort study, an elevated maternal NLR (indicative of systemic inflammation) was significantly associated with the development of NEC (19). It is critical to note that blood NLR is a key diagnostic and prognostic indicator for disease states such as diabetes, obesity, hypertension, and heart disease, which are marked by inflammation. As such, the positive association between elevated maternal NLR and NEC suggests a possible relationship between NEC and placental vascular dysfunction caused by these disease states.

Preeclampsia, a serious complication of pregnancy, is also associated with an increased risk of NEC in preterm infants. Although the pathogenesis of preeclampsia remains unclear, it is theorized that the placental ischemia, abnormal hemostasis, leukocyte activation, and dysregulated nitric oxide metabolism associated with preeclampsia seem to be core components that may contribute to NEC development in preterm infants (20). Overall, preeclampsia reduces placental perfusion, which can lead to fetoplacental hypoxia and the pathogenesis of intrauterine growth restriction (IUGR). Both IUGR and reduced placental support, as indicated by abnormal patterns in antenatal umbilical dopplers, can impose increased risks for later NEC development (20, 21). Additionally, maternal diabetes mellitus (DM) poses a significant risk of NEC to infants, as determined by a retrospective study of low birthweight infants born to mothers with and without DM (21, 22).

Birth route may also provide insight into an infant's risk of developing NEC due to the impact that birth route has on the infant microbiome. However, the effects of Cesarean section (C-section) on the risk of NEC development are highly contested. A recent retrospective review discovered that delivery by C-section (and the presence of an umbilical arterial catheter) is associated with a decreased risk of NEC, possibly due to a decreased stress burden on the neonate during the C-section birthing process as compared to vaginal birth (23). A secondary analysis of data from a randomized controlled trial found no significant association between C-section in extreme preterm delivery and the onset of NEC (24). In contrast, another national case-control study established a positive association between C-section and the risk of NEC (25). Thus, there is conflicting data describing the relationship between C-sections and NEC incidence in neonates. Such disparities in data further indicate that NEC is a multifactorial condition and additional studies are required to delineate the maternal conditions that may predispose an infant to the disease.

Infant prematurity, characterized by both low birth weight and gestational age, is one of the most important risk factors for the development of NEC. Several studies have established that infants with a lower gestational age have a greater risk of developing NEC, along with higher mortality and surgical need (26, 27). Another retrospective study reported a higher NEC incidence in preterm infants that are small for gestational age (SGA) (28). While NEC pathogenesis in SGA neonates has not been completely explained, it has been proposed that gastrointestinal (GI) tract ischemia can contribute to NEC pathogenesis in preterm infants. Immature development of the GI tract can prime a “leaky” gut barrier susceptible to bacterial translocation due to incomplete formation of tight junctions, impaired peristalsis, and a thin mucus layer (29). The reduced structural integrity of the gut barrier can further decrease the uptake of essential nutrients for growth, exacerbating the effects of NEC.

Different types of infant nutrition can impact the pathogenesis of NEC. The nutritional requirements of preterm infants usually cannot be sustained solely with breast milk or standard formula—bovine and human-milk-based fortifiers are often needed to provide additional proteins, fats, vitamins, and minerals for adequate growth and development. However, some studies suggest that bovine milk-based infant formulas are positively associated with a higher risk of NEC, reviewed in (30). Although the exact link between bovine milk-based standardized formulas and NEC pathogenesis is not clear, one theory suggests that in the absence of the protective factors found in breast milk, infants receiving formula are at an increased incidence of NEC. This may render the gut more susceptible to the overgrowth of pathogenic microbes, such as the family of Gram-negative Enterobacteriaceae, and the initiation of widespread pro-inflammatory responses to bacterial translocation across the gut barrier (31). In contrast, the administration of maternal breast milk has been conclusively established to decrease NEC incidence (32). It has been long-established that human milk is the ideal source of nutrition for both premature and full-term infants. Several studies have demonstrated that there is a clear benefit to maternal human milk or donor human milk in the absence of maternal milk, reviewed in (33). Premature infants who received human milk have a demonstrably lower incidence of NEC than those who did not (34).

Intestinal dysbiosis, or the imbalance of a healthy gut microbial composition, has also been implicated as a predisposing factor to NEC. It is known that the gut microbiome of preterm infants has considerably reduced bacterial diversity and increased vulnerability to pathogens as compared to full-term infants (35). Additionally, there is a positive association between early antibiotic use and NEC onset, which supports the intestinal dysbiosis hypothesis (36). There have also been reports of immune dysregulation in conjunction with intestinal dysbiosis, particularly concerning heightened toll-like receptor 4 (TLR4) signaling and downstream inflammatory responses (37). Taken together, the pathogenesis of NEC is multifactorial and complex, rendering the root pathophysiology of NEC largely unanswered.

## Immunological status of infants with NEC

**Figure 1:**
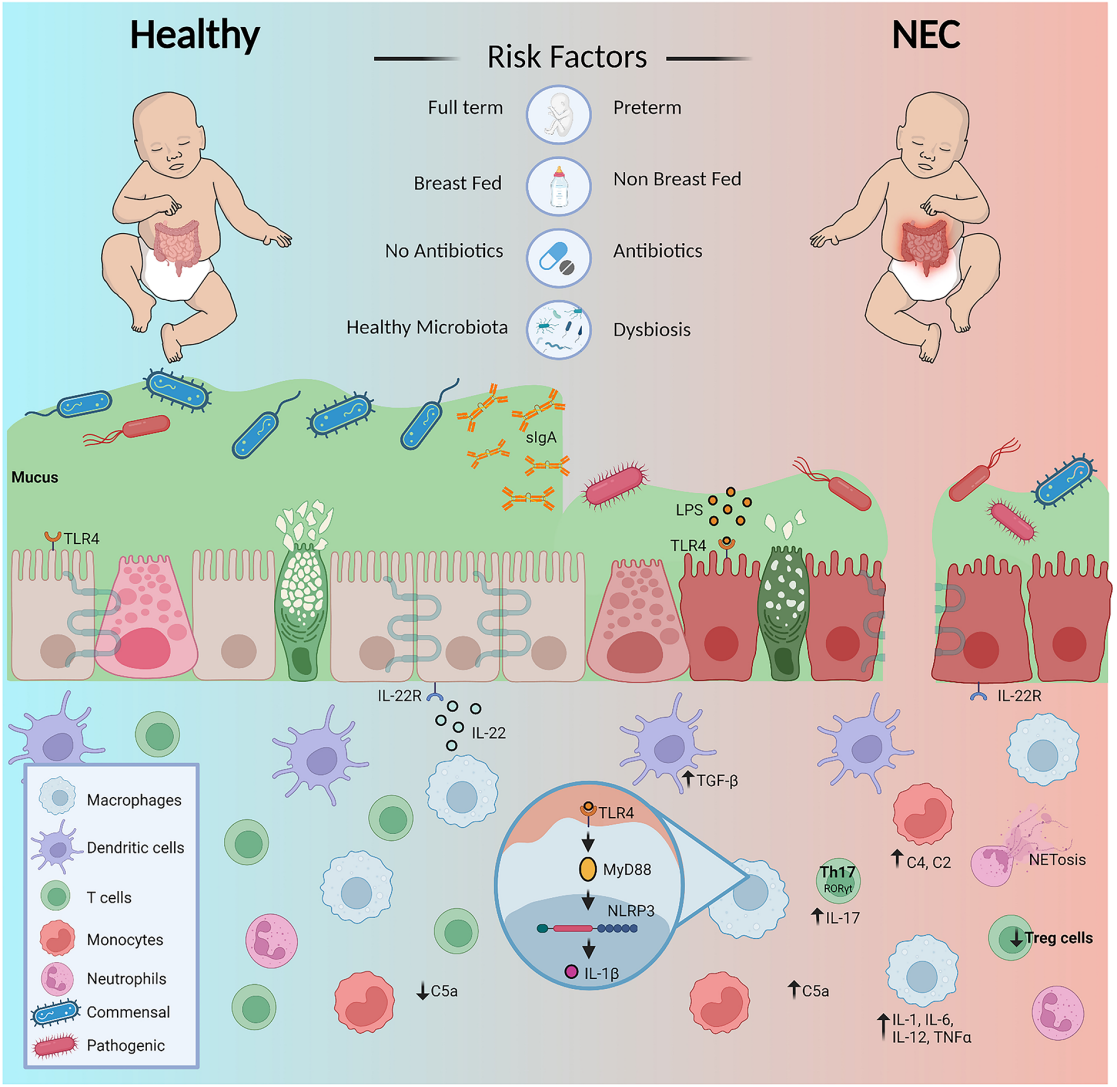
Diagrammatic overview of factors predispose premature infants to NEC and dysregulation of immunity contributing to the diseased state. Figure created with Biorender.com and affinity designer.

Immature intestinal immune defense is among several factors associated with the high morbidity and mortality rates of NEC. Alteration of key innate and adaptive immune responses leads to dysfunction in intestinal barrier thus resulting in an increased inflammatory response (Figure 1) (38–40). The onset of NEC has been linked to low birth weight and gestational age, so while all infants have immature innate immunity, premature infants are also born with undeveloped adaptive immune systems. To make up for this weakened immunity, the transfer of maternal milk components, including secretory IgA (sIgA), as well as placental immunoglobulin G (IgG), provide protection to newborns until their own adaptive immune defenses can develop (15). In formula-fed premature infants, the levels of transferred maternal immune defenses are significantly reduced, potentially increasing their susceptibility of developing NEC (41).

In this section, we summarize the current scientific evidence of the innate and adaptive immune responses in infants with NEC. Specifically, we discuss how NEC pathogenesis relates to the vertical transfer of immunity from mother to child, alteration in physical barriers, and immunity guarding the gastrointestinal tract.

### Maternal antibody transfer

Newborns do not cultivate a fully mature immune system until a few years after birth (42). To compensate, maternal IgG and IgA antibodies are donated from the placenta and maternal breast milk (if provided) to protect against pathogens and the development of NEC (15). Maternal IgG transfer to the fetus across the syncytiotrophoblast depends on the IgG-FcRn (neonatal Fc receptor) interaction. The expression of IgG-FcRn begins during the first trimester (12 weeks) of pregnancy and continues to rise until between 17 and 41 weeks gestation. The majority of placental IgG transfer occurs after 28 weeks of gestation. IgG levels reach 50% maternal concentration between 28 and 33 weeks gestation and will rise above maternal levels by 20%–30% at term (43, 44). It is possible that low IgG levels in preterm infants may predispose these infants to develop NEC.

In addition to the transfer of maternal IgG, transfer of maternal IgA through breast milk, also protects infants from NEC. Originating from IgA+ plasma cells in the gut and educated by gut microbiota, IgA in the intestine can bind to pathogens and aid in their clearance. The ability of bacteria to bind to IgA was negatively correlated to NEC development, and the reduced stool bacterial diversity known to precede NEC was associated with a higher amount of unbound *Enterobacteriaceae* (15). Taken together, this data suggests that the absence of sIgA creates higher susceptibility to infections as well as delayed gut microbiota maturation which leads to gastrointestinal inflammatory diseases such as NEC.

### Breast milk components

As the primary source of nutrition, breast milk ensures proper growth and development for newborns. Human milk is composed of micro and macronutrients, bioactive components, growth factors, antibodies, and HMOs (45). HMOs, in particular, play an important role in shaping microbiome composition and modulating neonatal immunity. HMOs act as natural prebiotics, functioning as soluble decoy receptors or antiadhesives to block the adhesion of pathogens to epithelium. They also enhance commensal growth and limit pathogen growth (46). HMOs are non-digestible sugars, composed of five basic monosaccharide units: glucose, fucose, d-galactose, N-acetylglucosamine, and sialic acid (47, 48). These monosaccharide units are joined by glycosidic linkage to generate a variety of HMOs with different functions. HMOs are indigestible in the human upper digestive tract and remain intact while in the colon. Colonic microbes secrete enzymes to utilize these HMOs as nutrition (49, 50). Many of the commensals that degrade HMOs for fuel are members of the Bifidobacterium genus, mostly beneficial bacteria for infant health. Specific examples are *B. longum* and *B. breve* that are usually prominent in the digestive tract of breastfed infants.

In addition, Bacteroides species possess an excellent capacity to metabolize dietary polysaccharides to host-derived mucus-associated glycans. A study by Sodhi and colleagues has shown that HMOs 2′-fucosyllactose (2′-FL) and 6′- sialyllactose (6′- SL) can reduce NEC severity through TLR4 inhibition (51). 2′-FL also suppresses lipopolysaccharide (LPS) induced inflammation during *Escherichia coli* (*E. coli*) invasion of intestinal epithelial cells (52). Similarly, Masi et al. found significantly lower disialyllacto-N-tetraose (DSLNT) in the maternal milk given to infants prior to NEC development (53). Furthermore, authors reported that low DSLNT in milk was also associated with a significantly lower relative abundance of Bifidobacterium sp. and higher *Enterobacter cloacae* in the stool of infants prior to NEC (53). Fractions of HMOs were also shown to decrease mucus penetrability and bacterial attachment by enhancing the expression of Mucin 2 (MUC2) in a mouse model of NEC (54).

Other milk factors such as casein, a highly glycosylated breast milk protein, promotes intestinal defenses by increasing goblet cell numbers, enhancing *Muc2* expression, and Paneth cell activity (55, 56). Additional factors found in breast milk include lactoferrin and lysozymes that possess antipathogenic properties. Enteral supplementation of lactoferrin has been shown to decrease the likelihood of late-onset bacterial and fungal sepsis in preterm infants, but meta-analysis has shown there was no significant decrease in NEC in infants who were received lactoferrin (16). Breastmilk platelet activating factor-acetyl hydrolase (PAF-AH) potentially protects preterm newborns from NEC (57). Similarly, interleukin-10 (IL-10) found in breast milk has been found protective against developing NEC in premature infants (58). In addition to IL-10, maternal transforming growth factor beta (TGF-β) provides protection by helping to increase IgA locally in the gut (59). Growth factors found in breast milk, such as insulin-like growth factor (IGF) and epidermal growth factor (EGF), support intestinal health and may protect against the development of NEC (60–65).

### First line defense of the intestinal barrier

Mucus is one of the first lines of intestinal host defense. Mucus is produced by goblet cells, which are found in the crypts of Lieberkühn. The colonic mucus layer is divided into two layers, an outer, penetrable layer, and an inner, impenetrable layer. This contrasts with the mucus in the small intestine (SI) which is single layered and penetrable by bacteria. A protective layer of mucus keeps bacteria in the SI away from the intestinal epithelium by antimicrobial proteins (AMPs) secreted by Paneth cells (66). Studies have found defective and a significantly lower number of goblet and Paneth cells in the SI of infants with NEC compared to NEC (67). Using HT29-MTX-E12, a mucus secreting cell line, Hall and colleagues reported that breast milk significantly lowered the adherence and internalization of NEC-associated pathogenic *E. coli* into the mucus compared to infant formula, suggesting that breast milk enhances mucus integrity (68). *Clostridium difficile (C. difficile),* a known gut pathogen, also influences mucus production and composition (69).

Antimicrobial peptides (AMPs), such as defensins, including human β-defensin-3 (hBD3), cathelicidins, C-type lectin receptors (CLRs), regenerating islet-derived protein 3, and intestinal enzymes such as phospholipase A2-IIA (PLA2) and lysozyme are expressed in the gut epithelium and provide protection for the intestinal mucosa from pathogenic bacteria either by killing pathogens or by inhibiting their growth (70, 71). In addition, AMPs are involved in the immune response and shaping the microbiome (72). Using an experimental rat NEC model, Underwood and colleagues found increased intestinal mRNA expression of the AMPs *lysozyme*, secretory *PLA*2, and *pancreatic-associated proteins* 1 and 3 in rats with NEC compared to either dam-fed or formula-fed rats supplemented with the probiotic bacteria *Bifidobacterium bifidum* (*B. bifidum*), suggesting that AMP induction is a mucosal response to gut inflammation in NEC (73). Another study evaluated the defensin hBD3, a small cationic antimicrobial peptide that can exert multiple protective properties on the gut. Using an animal NEC model, Sheng et al., showed that hBD3 administration decreased the incidence of NEC, reduced NEC severity (decreased pro-inflammatory cytokines, intact intestinal barrier), and increased the survival rate of the animals (74). Collectively, these studies suggest a protective role for mucus and associated AMPs in neonatal mucosal defense and intestinal barrier function in NEC.

### Complement proteins and NEC

During infection, complement proteins assist in the phagocytosis of invading pathogens by opsonization, generating inflammatory responses, and altering the activity of B and T lymphocytes (75, 76). Three different pathways—lectin, alternative, and classical—activate the complement cascade. Previous studies have reported defective complement protein activity in preterm infants (77, 78). More specifically, one study reported low complement component 3 (C3) and complement component 9 (C9), intermediates of complement pathways, in preterm infants (79, 80). C5a, a cleavage product of complement component 5 (C5), is a potent chemoattractant, anaphylatoxin, and intermediary in both the conventional and non-canonical complement pathways. C5a was reported to be substantially expressed in NEC cases and could be partially responsible for inflammation in NEC. Due to its multifaceted nature, C5a is being studied for its utility as a clinical marker for the diagnosis of neonates with NEC in conjunction with radiographic evidence of disease (81). In addition, MBL-associated serine protease-2 (MASP-2), an enzyme associated with C2 and C4 cleavage and activity, is detected in higher concentrations in the cord blood of premature children who are susceptible to NEC and is linked to a threefold increased risk of developing NEC (82, 83).

### Toll-like receptors and innate immune cells in NEC

*Drosophila* Toll was discovered as a receptor for dorso-ventral patterning during development and was later identified as a participant in immunity against fungal infections (84). Consequently, several other homologues of Toll, named Toll-like receptors (TLRs) were discovered in mammals. TLRs sense pathogen-associated molecular pattern molecules (PAMPs) and danger-associated molecular patterns (DAMPs) through their N-terminal extracellular leucine-rich repeats (LRRs) and elicit innate immunological responses, including the production and release of inflammatory cytokines (85). So far, 10 different types of TLRs have been identified in humans and 12 in mice. TLR1, TLR5, TLR6, and TLR10 are membrane receptors that may detect extracellular ligands while TLR3, TLR7, TLR8, and TLR9 work on subcellular structures. For example, TLR9 is found on endosomes and recognizes nucleic acids derived from pathogens and self-damaged cells (85, 86). TLR2 and TLR4 are expressed on the cell membrane as well as on subcellular structures.

TLR4 is a receptor for LPS, a component of Gram-negative bacteria's outer membrane that is critical for the NEC pathogenesis (87). TLR9 binds to and is activated by unmethylated cytosine-guanine oligodeoxynucleotides (CpG ODNs) in bacterial genomes, and acts as antagonist of TLR4. Activation of TLR4 in newborn mouse epithelial cells by LPS results in undesired activation of the NF-κB pathway that leads to damage of the intestinal mucosa through production of pro-inflammatory cytokines, which is one of the hallmarks of NEC (87). Several studies have shed light on the association of TLR4 with NEC (41). Recently, Liu and coworkers have shown both TLR4 and necro apoptotic protein upregulation in both NEC patients with NEC and animal NEC models (88). Egan et al., highlighted the role of TLR4 in recruiting the inflammatory CD4^+^ Th17 cells into the intestinal mucosa *via* activation of cognate chemokine ligand 25 (CCL25) in NEC (89). In an another study, Colliou et al., found a commensal *Propionibacterium* bacterial strain named UF1 that can reduce intestinal inflammation through the reduction of Th17 cell expansion in the gut of a mouse NEC model (90). TLR4 activation significantly inhibits the β-catenin signaling that is important for enterocyte proliferation in the ileum of newborn mice, which further leads to apoptosis and can lead to NEC (91). Studies have shown that activation of TLR9 can decrease experimental NEC severity, and that TLR9 activation can inhibit TLR4 signaling *via* IL-1R-associated kinase M (92, 93). In addition to TLR9, NOD2 reduces NEC severity *via* suppressing TLR4 and genetic variants in NOD2 are associated with NEC development (94, 95).

### Monocytes and macrophages

Originating from myeloid cell lineage monocytes, macrophages (Mϕ) act as a frontline guard of innate immunity against invading pathogens. Monocytes and Mϕ have several weapons in their arsenal to tackle incoming threats. By recognizing molecular patterns *via* toll-like receptors (TLRs), nucleotide-binding oligomerization domain-containing proteins (NOD2), and C-type lectin receptors (CLRs,) these cells either actively engage in phagocytosis or secrete various cytokines and chemokines to alert and recruit other immune cells (96). Classical monocytes (CD14^+^CD16^−^), intermediate monocytes (CD14^+^CD16^+^), and non-classical monocytes (CD14^dim^CD16^+^) are the three subsets of human monocytes. In mice, monocytes are grouped based on expression levels of lymphocyte antigen 6 complex (Ly6C^+^ and Ly6C^−^) on their cell surface (97).

Several studies have suggested that tissue infiltration and enrichment of monocyte-derived Mϕ occur during inflammation in NEC (98–100). Intestine monocyte-derived Mϕ are nonproliferative, short lived and terminally differentiated, rendering their continuous replacement necessary for homeostasis. A study by Managlia et al., revealed the significance of nuclear factor kappa B (NF-κB)-driven monocyte activation, recruitment, and differentiation in neonatal intestines in NEC (99). They concluded that NF-κB-mediated activation and differentiation of Ly6c^+^ monocytes into Mϕ and their recruitment into the intestine are critical for NEC development and disease progression. Olaloye and colleagues have identified a novel subtype of inflammatory CD16^+^CD163^+^ monocytes/Mϕ associated with infants with NEC (100). In infants with NEC, peripheral monocyte counts drop due to their recruitment to the damaged intestine (101). Following recruitment, monocytes undergo differentiation to form pro-inflammatory M1-type Mϕ (102). Monocyte-derived M1 Mϕ in humans and in animal models have been linked to the severity of NEC (102, 103). Interferon regulatory factor 5 (IRF5), a factor crucial for M1 Mϕ polarization is highly expressed in infants with NEC compared to controls. Specifically, IRF5 deficiency significantly reduced M1 polarization, inflammation, and intestinal injury in experimental NEC (103). Inflammation and intestinal cell damage caused by M1 Mϕ is linked with their high level of pro-inflammatory cytokines such as IL-1, IL-6, IL-12, chemokines (Ccl4, Ccl5), and tumor necrosis factor (TNF) production. By inhibiting M1 and promoting M2 polarization of Mϕ, heparin-binding epidermal growth factor (HB-EGF) has also been found to protect against experimental NEC (102).

### Neutrophils

As one of the most abundant immune cells (nearly 70% of total leukocytes) in human blood, neutrophils are among the first responders in the fight against potential pathogens or tissue damage/injury. Neutrophils eliminate pathogens either by recruiting a wide variety of immune cells through the secretion of cytokines, chemokines, and leukotrienes or by causing direct damage to tissue or pathogens by releasing lytic proteases and neutrophil extra cellular trap (NETs) (104). In addition to their well-documented protective role, neutrophils are also able to cause significant tissue damage through the release of reactive oxygen species (ROS) in intestinal inflammation (105).

Early neutropenia has been associated with higher odds of developing NEC (106). Interestingly, neutrophils in preterm newborns have altered immunological functions, including impaired phagocytosis. Another study by Zindl and colleagues revealed the protective role of IL-22-producing neutrophils in experimental colitis by increasing AMP production and promoting mucosal repair (107). In the context of NEC, a recent study from Mihi et al., demonstrated a protective role of IL-22 treatment in attenuating intestinal injury and enhancing epithelial proliferation in experimental NEC (108). This study also found that there was a lack of IL-22 production in preterm infants or developing mice, suggesting that immunomodulatory treatments may help protect premature infants from the intestinal inflammation seen in NEC.

As specialized antigen presenting cells (APCs), dendritic cells (DCs) serve as critical link between innate and adaptive immunity. In intestine, DCs are present in Peyer's patches, mesenteric lymph nodes (MLNs), and the colonic lamina propria to provide protection against invading pathogens. To date, several studies have highlighted the protective role of DCs in regulating the gut inflammation; however, studies investigating the role of DCs in NEC is limited. In one study, which utilizes *Cronobacter sakazakii* (*C. sakazakii*) to induce NEC in mice, Emami and colleagues have reported higher DC recruitment in mouse gut. They found that DC recruitment to the gut accelerated the destruction of the intestinal epithelium and promoted NEC onset with increased TGF-β production (109). *C. sakazakii* also induced pyroptosis in the intestinal epithelium and promoted NEC by induction of IL-1β and Gasdermin D (GSDMD) through TLR4/MyD88 mediated activation of the nucleotide-binding oligomerization domain (NLRP3) inflammasome (110). Another study by Nolan and colleagues investigated the role of aryl hydrocarbon receptor (AhR) signaling in DCs during experimental NEC, as this signaling pathway helps regulate intestinal immunity and homeostasis. They found that a lack of AhR signaling in DCs increased NEC-mediated intestinal inflammation, and that this effect was associated with an increase in a specific subset of macrophages in the small intestinal lamina propria (111).

### Trained immunity and NEC

Adult human intestine is made of a single layer of epithelium, covering an area of 32 m^2^ (112). The intestinal epithelium is important for digesting food and absorbing nutrients, but it is also the largest entry port for pathogens. To provide protection against these pathogens, “as a guard of port”, complex and tightly controlled innate and acquired immunity are required. Among the many different types of immune cells involved in this protection are intraepithelial lymphocytes (IELs). IELs are positioned between intestinal epithelial cells and constantly patrol the epithelial barrier (113). IEL subsets, composed of antigen-experienced T cells, are in direct contact with enterocytes and antigens in the gut lumen. These cells are classified based on the expression of T cell receptor-γδ (TCRγδ)^+^ and TCRαβ^+^ (114). Approximately 60% of small intestinal IELs are TCR^+^ cells. γδ IEL play a crucial role in mucosal defense by regulating the production of IgA, clearing and repairing damaged epithelium, increasing production of TGF-β cytokines and by decreasing IFN-γ and TNF-α in response to stress and infection (115). The protective role of IELs is also evident in TCRγδ-deficient mice, as these mice have defective gut epithelial morphology and impaired IgA production (116). When compared to non-NEC controls, surgical NEC patients with NEC had a lower number of γδ IELs in the ileum (116). Researchers have shown that subsets of IELs are dependent on AhR activation for their survival (117). However, a recent study did not find any involvement of IELs in AhR activation-mediated protection against NEC, indicating that the protective role of IELs against NEC is not AhR-mediated (118).

In addition to IELs, infants with NEC also have altered functions of some subsets of CD4^+^ T cells, Th17, and regulatory T (Treg) cells (89, 119–121). Th17 cells are strongly implicated in intestinal inflammation and are linked with the pathogenesis of NEC. In infants with NEC, Pang and colleagues found a lower percentage of Foxp3-expressing Tregs with several functional defects, including the inability to block IL-17 expression (121). In NEC tissue, Th17 cells appear to cause intestinal damage that is reduced by IL-17 receptor inhibition by STAT3 activation (122). Additionally, retinoic acid-induced polarization of CD4^+^ T cells towards Treg from Th17 resulted in reduced NEC severity (123). Furthermore, Zhao et al. reported an increased percentage of RORγt^+^ cells (inflammatory Th17 and type 3 innate lymphoid cell populations) in the intestinal lamina of mice and humans with NEC compared to those without NEC (84). Studies have also demonstrated a significant decrease in lamina propria associated Treg cells in surgical NEC specimens (85, 86, 89). In addition, a Treg/Th17 imbalance leads to the excessive proinflammatory response preceding tissue injury and necrosis associated with NEC development (122).

## Intestinal microbiome and NEC

Although the direct association between the microbiota and the pathogenesis of NEC is not well understood, mounting evidence suggests a link between early gut microbiota dysbiosis and NEC (87, 88, 90). Probiotic supplementation to premature neonates has been shown in some studies to decrease the incidence or severity of NEC, further establishing the relationship between NEC and microbiota (91–94).

Early microbiota composition and its diversity in the gut of newborn infants is mainly influenced by delivery mode, antibiotic exposure, human milk feeding, and time spent in the NICU. Vaginally born infants not only develop stronger immunity but also are predominantly colonized by beneficial microbes such as *Lactobacillus* sp. present in mother's vaginal microbiota (95). Members of *Lactobacillus* are well known to prevent pathogen colonization by lowering the pH or by secreting inhibitory compounds (124, 125). The microbiota of infants born *via* C-section resemble the mothers' skin microbiota in early life and lack members of *Bacteroides* species that are present mostly in vaginally-delivered infants (126).

In addition to delivery mode, feeding also affects microbiome composition and diversity. Formula-fed newborns have lower overall bacterial diversity, lesser beneficial bacterial number, and a higher number of pathogenic bacteria like *Clostridium* sp. compared to breast-fed infants (127). *Clostridium* sp*.* and their secreted toxins can be associated with NEC severity (128, 129). Time spent in the NICU with lifesaving machines attached to preterm infants including, ventilators, and incubators, have also been shown to harbor pathogenic bacteria including members of *Streptococcus*, *Klebsiella*, *Staphylococcus*, *Neisseria*, and *Enterobacteriaceae* communities (130–133). Members of the phyla Firmicutes, such as coagulase-negative staphylococci (CoNS) and Proteobacteria are implicated in NEC pathogenesis, however, many of their members are also found in healthy infants (134). Higher bacterial relative abundance from the class Gammaproteobacteria, namely *C. sakazakii*, *Klebsiella* sp., *E. coli*, and those from the phylum Proteobacteria are also present in the feces of infants who develop NEC (135). In addition to bacteria, viral presence is also associated with NEC. Stool analysis from 51 infants with NEC and 39 controls demonstrated that the presence of adenovirus and Epstein-Barr virus are associated with NEC severity (136). In another recent study, stool samples obtained from 9 infants with NEC infants and 14 controls matched for weight and gestational age, showed reduced viral beta diversity over the 10 days before NEC onset. This study also identified that viral NEC-associated contigs belonging to *Myoviridae*, *Podoviridae* and *Siphoviridae* are associated with the time period 0–10 d post NEC onset (137).

## Models for studying NEC

### In vivo

With the high prevalence of NEC, the need for effective *in vivo* models has become more important in recent years. Due to the aggressive nature of the disease and the scarcity of available human specimens, performing experiments with human samples is difficult and multi-center studies are typically needed (138). As a result, animal models are commonly used to study NEC by inducing inflammation that mimics the intestinal damage seen in human infants.

**Figure 2:**
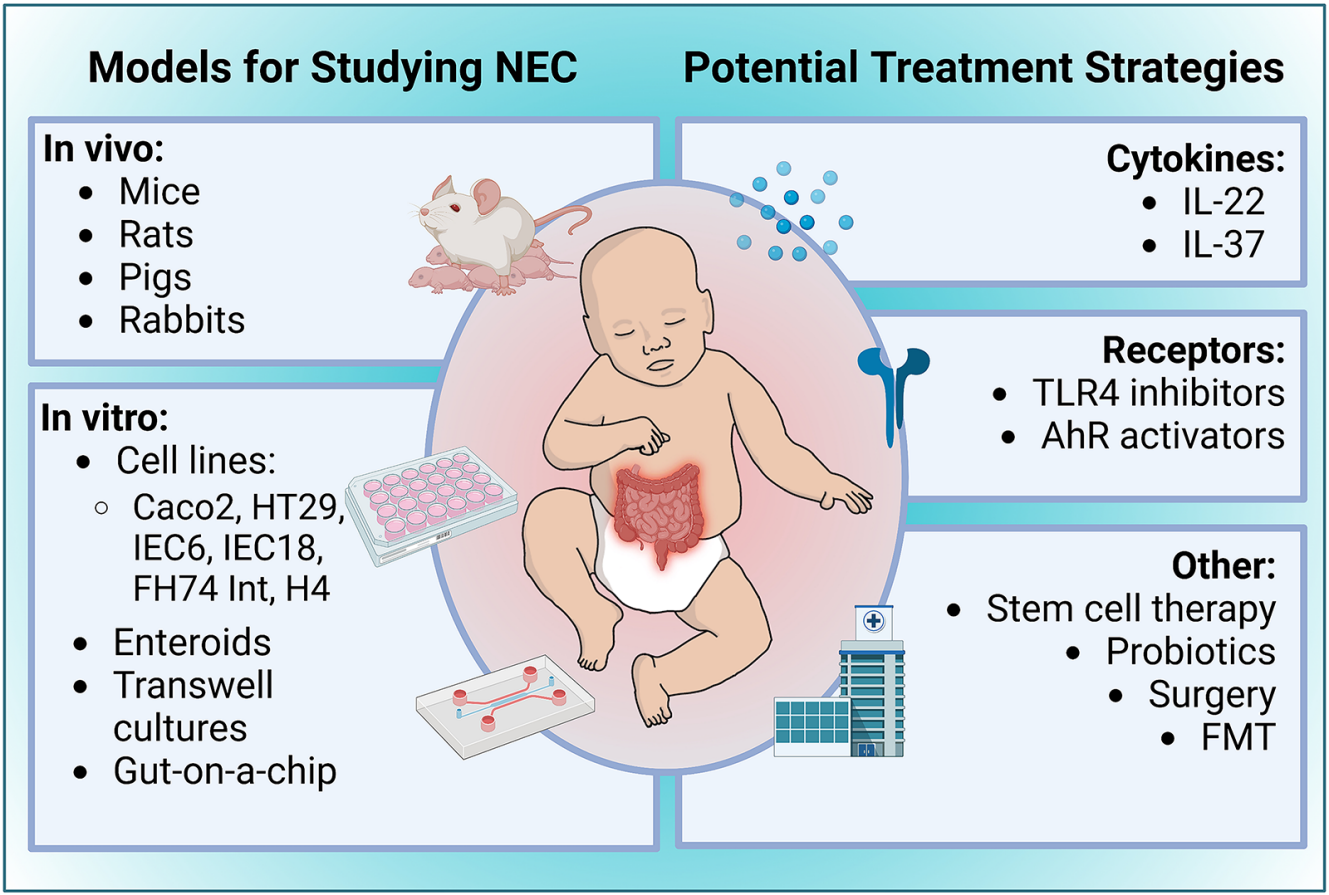
Overview of experimental models of NEC and potential treatment strategies. Figure created with Biorender.com.

While the conditions of *in vivo* experimental NEC models are generally based on similar underlying principles, several different animals have been used to study NEC (Figure 2). The rat's intestinal development is similar to a human premature infant, making it an excellent model for investigating preventative measures and therapeutics for NEC (139). Early studies using a rat model concluded that the gut microbiota and the absence of breast milk are significant factors in NEC pathogenesis (140, 141). Further, several laboratories have used hypoxia, LPS, and hypothermia at different time points in a day for several days to help induce NEC in laboratory settings (142). Due to their affordability, preterm survivability, and resistance to typical stressors used to develop the disease, rat models are a desirable option when investigating NEC but rats are not ideal for research at the genomic level. Their slower development and challenges with culturing embryonic stem cells in rats makes it difficult to generate transgenic lines compared to mice (143, 144). These shortcomings necessitated the creation of other types of animal NEC models.

Although their small size makes them technically challenging to work with, mice are the preferred model for genomic studies as it is far easier to create transgenic colonies. Another appealing feature of the mouse model is its experimental flexibility, with some models successfully inducing NEC by beginning the gavage feed at postnatal day 4 while others begin at postnatal day 7 (145, 146). However, mice delivered more than one day prior to the determined due date have a 100% mortality rate (147). Because of this low viability, it is extremely difficult to use a preterm mouse model for studies that require animals to be delivered *via* cesarean section.

Pigs share many features of anatomy and physiology with humans, rendering them one of the more popular choices when exploring NEC pathogenesis. Additionally, the piglet's larger size affords the ability to study preterm neonates (148, 149). Piglets are a good model for testing preclinical drugs, effects of various diet formulations, and pathological manifestation on NEC (150). While it is true that hypoxia and hypothermic stress induces histological changes that resembled NEC in piglet models, the inflammation induced by this model is not always contained within the lower gastrointestinal tract, with some instances reported of inflammation spreading to the stomach and jejunum (139, 150–152).

Rabbit NEC models are infrequently used but have been used to study the effects of NEC that extend past the gut. Non-human primate models, although rare and expensive, have also been used as an experimental NEC models due to the homology to humans in both anatomy and at the genomic level (139).

### In vitro

*In vivo* animal models allow for limited NEC modeling as the cellular genetics, drug metabolism, immunology, gut microbiomes, and HMOs can differ significantly from humans. *In vitro* intestinal models used to study NEC are briefly summarized in this section and have been covered extensively elsewhere (153–156).

Different *in vitro* models such as the human epithelial cell line Caco2, colon adenocarcinoma derived cell line HT-29, IEC-6 and IEC-18 derived from rat SI, and most importantly, fetus derived FHs 74-Int and H4 cells are frequently used in *in vitro* NEC studies (153). These cell lines are optimized and phenotypically mimic different regions of the gut including ileum, duodenum, jejunum, and colon, each requiring specific culturing conditions.

Recent scientific advancements in culturing human intestinal organoids (enteroids) also called “mini guts”, allow investigators to recapitulate the intestinal cell morphology that is crucial for studying the molecular mechanisms of NEC. Enteroids derived from LGR5^+^ progenitor cells of the SI and colon, allow for the study of barrier function, gut inflammation, cell proliferation, drug responses, and intestinal microbial interactions characteristic of NEC (157). Further advancements of *in vitro* models led to the development of a “gut-on-a-chip”, a method which cultures intestinal cells to mimic the microenvironment of the intestine (158, 159). The gut-on-a-chip model provides a suitable environment to culture different human cell types including epithelial, endothelial, and immune cells with gut microbes together in a controlled environment, to explore gut physiology and inflammatory changes seen in NEC, and can also be used as a pharmacological platform to test potential drug treatments (160).

Though, these *in vitro* models excellently resemble human intestine, several key criteria are considered in cell culture model design. Table 1 compares common different models and devices, specifically summarizing whether the models are static or microfluidic, *in vitro* or *ex vivo,* cell differentiation, cell polarity (apical out or basal out), nutrient absorption, drug metabolism, crypt villus formation, mechanical stimulation or peristalsis, oxygen gradient modulation, measure trans epithelial electrical resistance (TEER), co-culture with endothelial, vascular, and immune cells, and co-culture with gut microbes.

**Table 1:** Characteristics and limitations of *in vitro* static and microfluidic devices for NEC disease modeling (• = yes, *ο* = no).

Model	Description	Nutrient absorption	Co-culture	Differentiation	Drug metabolism	Microbiome	Crypt-villus axis	Oxygen gradient	Mechanical stimulation	Fluid flow	NEC modeling	TEER	Model Advantages	Model Disadvantages	References
*Static*															
Transwell	A 2D dual chamber well separated by a porous membrane allowing for compartmented cell culture media, cells, and drugs.	●	●	○	○	●	○	○	○	○	●	●	Simple multi-well culture model, can be modified for differentiation, endothelial co-culture, and immune cell migration.	Microbiome interactions are limited due to static culture (<24 h). Rapidly becomes overgrown.	(161, 162)
Organoid	An expanded 3D-spherical cell culture from intestinal LGR5^+^ stem cells (enteroids). Organoids are differentiated to resemble intestinal epithelial tissue in a 3D matrix.	●	○	●	●	●	●	○	○	○	●	○	Can be expanded and differentiated with apical or basal polarity in ECM. Suitable for assays. Can form villus-like structures.	Cannot be co-cultured with endothelial cells. Static culture becomes easily overgrown by microbes (<1 h).	(163–166)
Ex vivo	Functional live tissues with complex cellular components that replicate *in vivo* environments.	●	○	●	●	●	●	●	○	○	●	●	Complex differentiated tissue most similar to *in vivo* tissue.	Limited by tissue availability. Static microbial culture (<3 h).	(167)
Scaffold	An artificial intestine that mimics native intestinal architecture. Stem cells are seeded onto the scaffold and differentiated to form villus-like structures.	○	○	●	○	○	●	○	○	○	○	○	Provides a structured scaffold for crypt-villus formation. Enhanced metabolic enzymatic activity relative to 2D-cultures or chips without scaffolding.	Cannot be co-cultured with endothelial cells. Not suitable for microbiome co-culture. No basal permeability.	(168)
*Microfluidic*															
Ex vivo	Live functional intestinal tissue section enclosed in a microfluidics chamber.	●	●	●	●	●	●	○	●	●	●	●	Live functional tissue is subject to microfluidic flow where tissue is differentiated with crypt-villus structures and supportive endothelial tissues.	Requires fresh tissue. Very short time frame (<3 h) for tissue viability.	(169, 170)
Multichannel	A PDMS microchannel system (HuMix) with 3 co-laminar fluidic channels. An epithelial, medium perfusion and a microbial culture channel. The microbe channel is separated from the epithelial layer by a nanoporous membrane (0.5–1 mm).	●	●	○	●	●	●	●	○	●	○	○	Designed for TEER measurements and oxygen gradients across multiple channels. Allows for a membrane separated microbial and epithelial chamber to reflect microbial/cell signaling in a healthy gut.	Bacteria separated from the epithelial cells. No mucus layer interaction. Intentionally not designed for direct bacterial interaction and bacterial movement across the epithelial barrier required for NEC studies.	(171)
Gut-on-a-Chip	A PDMS dual microchannel system designed for specific gastrointestinal tissues. Gut-on-a-chip microfluidics are designed for 3D differentiated tissue.	●	●	●	●	●	●	●	●	●	●	●	Intestinal epithelial cells or organoids are cultured under peristalsis as a differentiated layer on an ECM scaffold. Continuous flow allows for extended culture (>7 days) and increased differentiation. Allows for co-culture with endothelial cells and a NEC microbiome. Can be cultured under different oxygen conditions.	Requires a high operating cost/chip and dedicated equipment. Requires enteroids. Experiments have a longer turn-over time and may require >7 days to allow for confluence and differentiation. PDMS may absorb small molecules.	(154, 172–174)

### Static vs. Microfluidic models

Static models are standard tissue culture models which include “NEC-on-a-dish” 2D, 3D organoid and transwell culture models (175). Additionally, synthetic scaffolds, and *ex vivo* tissue (Ussing chambers) are used to measure live tissue (167, 168). Static models use growth factors to differentiate intestinal epithelial cells (IECs) and organoids, derived from LGR5^+^ progenitor cells, into diverse functional intestinal cells (163). Static models are generally less time consuming, less expensive, and more accessible, but are relatively limited to the degree of differentiation, co-culture, and microbiome interactions. Typically, in static models, microbiome interactions are limited to between 1 and 24 h based on the model due to rapid microbial overgrowth in static conditions.

Gut-on-a-chip microfluidic devices use soft lithography to layer polydimethylsiloxane (PDMS) or micromilling to produce luminal and vascular channels separated by a porous membrane (reviewed in (176). Short term *ex vivo* microfluidic devices can evaluate live tissue conditions under constant flow (169, 170). The luminal flow in a microfluidic model enhances differentiation and 3D villus and crypt-villus like topography where adjacent air channels are regulated to mimic peristalsis through mechanical stimulation, thus providing a major advantage over static models. The NEC microbiome and HMO interactions, drug metabolism, and tissue integrity assays can be measured within the microfluidic chip system (177, 178). A major advantage of the microfluidic flow is that it reduces the static overgrowth of microbes, in turn reducing the limitations on the microbial co-culturing time to more than 7 days, depending on the specifics of the model. Gut-on-a-chip models can additionally be cultured under oxygen gradient modulation. Intestinal disease pathology is increased by lower oxygen gradients which induce Hif1-α signaling (179). Oxygen gradients under aerobic, hypoxic, and anaerobic culturing conditions have also been applied to resemble microbial intestinal environments under inflammatory conditions (176).

## Treatments for NEC

The several known risk factors of NEC discussed in this review provide promising treatment targets for NEC (Figure 2). One such treatment is IL-22, a cytokine belonging to the IL-10 family that is involved in epithelial cell regeneration, maintenance of gut barrier integrity, and tempering intestinal inflammation by mediating the microbiome (180). Given the observations of the versatile roles that IL-22 plays in gastrointestinal physiological processes and pathologies, especially as a stabilizer of intestinal homeostasis, there is a strong foundation to investigate the role of IL-22 in the context of NEC pathogenesis. As mentioned above, a recent study by Mihi et al., showed that neonatal mice and humans lack intestinal IL-22 production during NEC and supplemental administration of IL-22 attenuated experimental NEC severity, decreased intestinal inflammation, and enhanced intestinal epithelial repair (108). Additionally, IL-22 administration induced the expression of antimicrobial genes such as *Reg3γ* and fucosyltransferase 2 (*Fut2*). The AMP Reg3γ has been shown to protect the intestinal mucosa against pathogenic infections by limiting their expansions. Given this protective role of IL-22 in the experimental murine model of NEC, it is imperative that IL-22 administration be further investigated as a therapeutic for infants with NEC (108).

Another study by Cho et al., highlighted the importance of another cytokine, IL-37 in attenuating the inflammation in NEC (181). The study found that transgenic IL-37 pups were completely protected from inflammation caused by IL-1β, IL-6, TNF, and IL-17F compared to wild-type mice. In addition, IL-37 treatment restored the expression of cytokines *Il4*, *Il13*, and *Il33* to baseline levels. Further, authors found that IL-37-mediated protection against NEC is largely achieved through modulation of the TLR repertoire (reducing TLR4 expression and inducing TLR5, TLR7, TLR9, and TLR13), and prevention of NEC-induced dysregulation of adaptive immunity (181).

Another promising treatment modality is the use of TLR4 inhibitors to mediate intestinal injury propagated by NEC. Hackam and colleagues have published several studies indicating that expression of TLR4 and members of its gene family render the premature intestine more susceptible to inflammation. Therefore, exploring TLR4 modulation or inhibition as a model for NEC treatment may be valuable. Lien et al., and Tidswell et al., noted the synthetic inhibitor eritoran tetrasodium (E5564) bound well to TLR4 (182, 183). Based on the structure of this inhibitor, an *in silico* search and screening of small molecule libraries conducted by Hackam and colleagues pinpointed a family of TLR4 inhibitors that reduces intestinal inflammation in experimental NEC (184, 185). Particularly, the compound C17H27NO9 (C34), a 2-acetamidopyranoside, significantly reduced NEC incidence in animal models and decreased TLR4 signaling *ex vivo* in resected ileum from infants with NEC (185). Indeed, these findings indicate C34 and its analogs are lead compounds for TLR4 inhibition that can provide therapeutic value and improve clinical treatments for NEC. In a recent study Lu et al., showed that activation of AhR either by its ligand indole-3-carbinol or by breast milk components prevented experimental NEC through inhibition of TLR4 signaling (118).

Stem cell therapy is another treatment option currently being explored because of anti-inflammatory properties with a focus on bone marrow-derived mesenchymal stem cells (BM-MSCs). Several studies have demonstrated that BM-MSCs extracted from mice, rats, and humans significantly reduce both NEC incidence and severity (186–188).

Amniotic fluid-derived stem cells (AF-MSCs) have also been investigated as a potential source for NEC treatment. A study by Zani et al., established that intraperitoneal injections of AF-MSCs in a murine model are significantly associated with a reduction in the incidence and severity of NEC and improved gut barrier function (5). Subsequent confirmatory studies verified that AF-MSC injections decrease histologic injury in experimental NEC models (189). Thus, there is indication that AF-MSCs have considerable beneficial effects as an inflammatory modulator and should be examined further as a therapeutic for NEC.

Experimental results of supplementation with probiotics and potentially fecal microbiota transplant (FMT) has also shown promising outcomes to treat NEC, however, appropriate donor selection, screening of FMT material, and a dosing strategy still need to be standardized (190–192).

## Conclusion

NEC is a common gastrointestinal disease in premature infants associated with high morbidity and mortality. In recent years, substantial progress has been made to delineate the molecular mechanisms underlying the pathogenesis of NEC. The holistic approaches with scientific advancement to understand the risk factors predisposing an infant to NEC, including maternal, genetic, nutritional, and immunological changes in infants, clearly hold the potential to improve and lead to development of preventative measures and treatments to combat NEC. Although translating fundamental experimental discoveries to the bedside in the NICU is substantially challenging, continuous scientific efforts and collaborations between those working “at the bench” making discoveries in laboratories with those clinicians “at the bedside” caring for infants with NEC can lead to ground-breaking discoveries and transform the management of this devastating disease.
